# Frontiers in Three-Dimensional Surface Imaging Systems for 3D Face Acquisition in Craniofacial Research and Practice: An Updated Literature Review

**DOI:** 10.3390/diagnostics14040423

**Published:** 2024-02-14

**Authors:** Pradeep Singh, Michael M. Bornstein, Richard Tai-Chiu Hsung, Deepal Haresh Ajmera, Yiu Yan Leung, Min Gu

**Affiliations:** 1 Discipline of Orthodontics, Faculty of Dentistry, The University of Hong Kong, Hong Kong SAR, China; pradeepal1928@gmail.com (P.S.); deepal@hku.hk (D.H.A.); 2 Department of Oral Health & Medicine, University Center for Dental Medicine Basel UZB, University of Basel, Mattenstrasse 40, 4058 Basel, Switzerland; michael.bornstein@unibas.ch; 3 Department of Computer Science, Hong Kong Chu Hai College, Hong Kong SAR, China; richardhsung@chuhai.edu.hk; 4 Discipline of Oral and Maxillofacial Surgery, Faculty of Dentistry, The University of Hong Kong, Hong Kong SAR, China; mikeyyleung@hku.hk

**Keywords:** three-dimensional surface imaging, 3D face acquisition, 3D facial scanning, surface scanning, face scanning, face acquisition systems

## Abstract

Digitalizing all aspects of dental care is a contemporary approach to ensuring the best possible clinical outcomes. Ongoing advancements in 3D face acquisition have been driven by continuous research on craniofacial structures and treatment effects. An array of 3D surface-imaging systems are currently available for generating photorealistic 3D facial images. However, choosing a purpose-specific system is challenging for clinicians due to variations in accuracy, reliability, resolution, and portability. Therefore, this review aims to provide clinicians and researchers with an overview of currently used or potential 3D surface imaging technologies and systems for 3D face acquisition in craniofacial research and daily practice. Through a comprehensive literature search, 71 articles meeting the inclusion criteria were included in the qualitative analysis, investigating the hardware, software, and operational aspects of these systems. The review offers updated information on 3D surface imaging technologies and systems to guide clinicians in selecting an optimal 3D face acquisition system. While some of these systems have already been implemented in clinical settings, others hold promise. Furthermore, driven by technological advances, novel devices will become cost-effective and portable, and will also enable accurate quantitative assessments, rapid treatment simulations, and improved outcomes.

## 1. Introduction

Digitizing dental care is a contemporary approach to achieving optimal clinical results. The evolution of digitization in dentistry has been fueled by cutting-edge technologies, particularly in three-dimensional (3D) surface imaging, and is progressing towards digital treatments. Three-dimensional facial images reflecting actual faces are deemed the most reliable for detecting, planning, and predicting treatment outcomes [1]. Ongoing advancements in 3D surface imaging have enabled surgeons and orthodontists to assess surgical outcomes and gather detailed information about craniofacial structures [2,3]. Moreover, the current 3D surface-imaging technologies offer more detailed information and zero ionizing radiation than conventional imaging methods [4]. With the advent of 3D surface-imaging systems, a variety of clinical applications are now possible [5,6], including virtual articulation [7], obstructive sleep apnea diagnosis and prediction [8], smile design [9], virtual patient simulation [10], augmented-reality-driven real-time visualization of an operative scenario [11], and even interdisciplinary communication [12,13]. Also, the integration of 3D facial scans with artificial intelligence (AI) and machine learning (ML) [14] has made it possible to devise patient-specific treatment plans for plastic and reconstructive surgery [15], in addition to automating diagnosis in maxillofacial surgery and the identification of children with Autism Spectrum Disorder (AUD) [16]. Thus, there is a growing interest in adapting these technologies for various medical and dental disciplines.

An array of 3D surface-imaging technologies, including stereophotogrammetry, laser-based scanning, and structured light scanning, are currently available for generating photorealistic 3D facial images; however, 3D face acquisition systems based on these technologies differ in their accuracy, reliability, resolution, usability, and portability [17]. While the incorporation of some cutting-edge systems in a clinical setting without an in-depth understanding of the technicalities and their application could be an arduous task, others may be unsuitable because of constraints on resources, time, or space. Therefore, choosing a purpose-specific 3D face acquisition system could be a challenging task for clinicians owing to varied costs and hardware and software characteristics. Practitioners would feel more at ease implementing 3D surface imaging technologies into the dental workflow if they comprehended the various characteristics of the 3D face acquisition systems. Hence, given the breadth of incessantly evolving 3D surface imaging technology, providing clinicians and readers with updated information analyzing the various 3D face acquisition systems that are currently available is indispensable. Although several reviews of 3D surface imaging systems have been published previously [3,18,19], the majority are technical and engineering-specific. Therefore, with a focus on 3D face acquisition, this review aims to provide clinicians and researchers with an overview of the 3D surface imaging technologies and systems that are currently being used or have the potential to be used in the future for 3D face acquisition in craniofacial research and daily practice. The information presented in this review will guide clinicians in selecting an optimal 3D face acquisition system that is best suited for their clinical environment.

## 2. Materials and Methods

### 2.1. Eligibility Criteria

**Figure 1:**
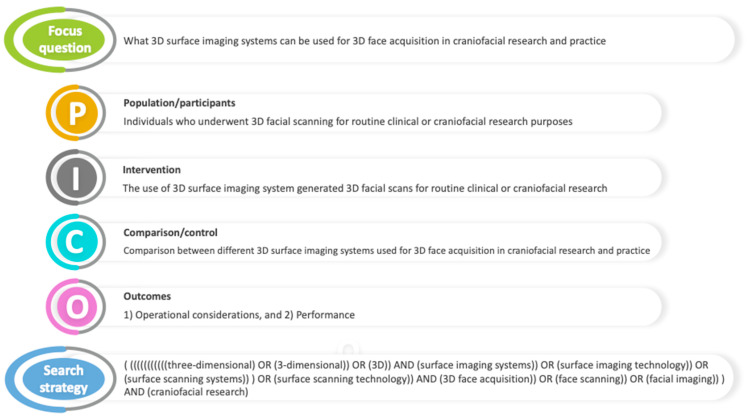
Description of the PICO (P = population/participants; I = intervention; C = comparator/control; O = outcomes) elements used in structuring the research question and the search strategy.

A literature search was conducted to find studies that matched the *Population–Intervention–Control–Outcome (PICO)* criteria for the topic: “What 3D surface imaging systems can be used for 3D face acquisition in craniofacial research and practice?” Only studies that addressed this question were considered appropriate for this review. The PICO criteria elements can be found in Figure 1.

The inclusion criteria for the studies were as follows: (1) utilization of a 3D surface imaging system for craniofacial research; (2) conducted on humans with proper analytical design (e.g., case–control studies, cross-sectional studies, prospective studies, retrospective studies including pilot studies); (3) absence of data duplication or overlap with other articles; and (4) availability of full-text studies published in English. Studies involving animals, those not focused on craniofacial research, those lacking data on hardware or software characteristics, those failing to report the type of 3D surface imaging system used, letters to editors, and review articles were excluded from this review.

### 2.2. Information Sources and Literature Search

Two authors (P.S. and D.A.) conducted a systematic and independent search for relevant studies. The search was comprehensive and included the electronic databases PubMed, EMBASE (via Ovid), Medline (via Ovid), Cochrane Library, Scopus, and Web of Science. The search spanned from January 2008 to November 2023 and utilized a combination of medical subject heading (MeSH) terms as keywords. Adjustments were made to the vocabulary and syntax across the databases. The search was not limited to published studies. In addition to the computer-assisted search, manual searches were conducted in scientific journals, online literature, reference lists of relevant reviews and retrieved studies, and articles that may have been missed. Furthermore, unpublished data were sought through the OpenGrey database, http://www.opengrey.eu/ (accessed on 7 November 2023).

### 2.3. Study Selection

Following a comprehensive literature search, two authors (P.S. and D.A.) systematically evaluated the titles and abstracts of potential studies to determine their eligibility based on predetermined inclusion and exclusion criteria. Any disagreements regarding inclusion were resolved through discussion. If disagreements persisted, a third author (G.M.) independently evaluated the articles. Full-text studies that met the inclusion criteria were retrieved. Endnote™, version 20 (Clarivate Analytics, Philadelphia, PA, USA), was used for collating, managing potentially eligible records, and obtaining bibliographic citations from the literature search.

### 2.4. Data Extraction and Outcomes of Interest

The two reviewers (P.S. and D.A.) independently performed data extraction using a standardized and predefined data format. The aim was to discern the following outcomes of interest: (1) operational considerations and (2) performance. To achieve this, relevant data from the full-text articles were extracted as follows:

(1) Hardware characteristics (portability, system mobility, sensor position, cost-effectiveness);
(2) Software characteristics (CT/CBCT integration, surgery simulation, real-time 3D volumetric visualization, tissue behavior simulation, progress and outcome monitoring);
(3) Functionality (purpose, data delivery, capture speed, processing time, scan range, coverage, optimal 3D measurement range, color image, scan requisite, output format, scan processing software enabled, accuracy, precision, archivable data, user-friendliness, system requirements, calibration time). Table 1 illustrates the definitions of various characteristics studied in 3D face acquisition systems.

The study is organized into several sections. Section 3 delves into 3D surface imaging technologies and systems. Section 4 provides a discussion of general considerations and practical information about 3D face acquisition. Section 5 presents future directions. Lastly, the final section provides conclusions drawn from the study.

## 3. Results

**Figure 2:**
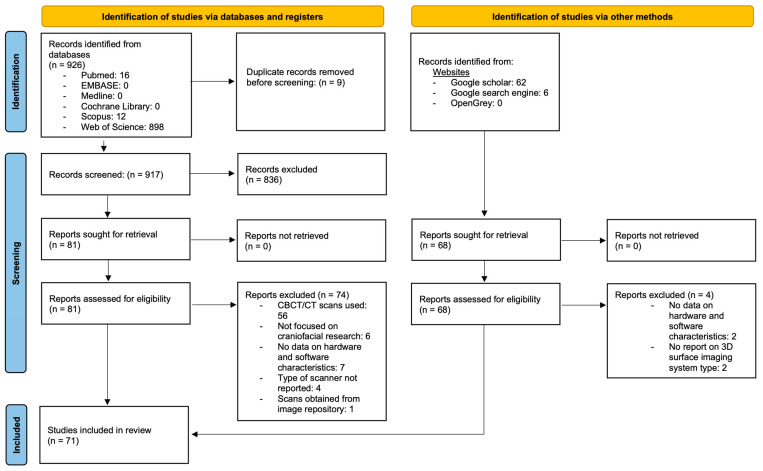
Flow diagram illustrating the study selection process.

Figure 2 presents the flowchart of the study selection process. A comprehensive search of six databases initially identified 926 records, and an additional 68 records were found from other sources.

**Figure 3:**
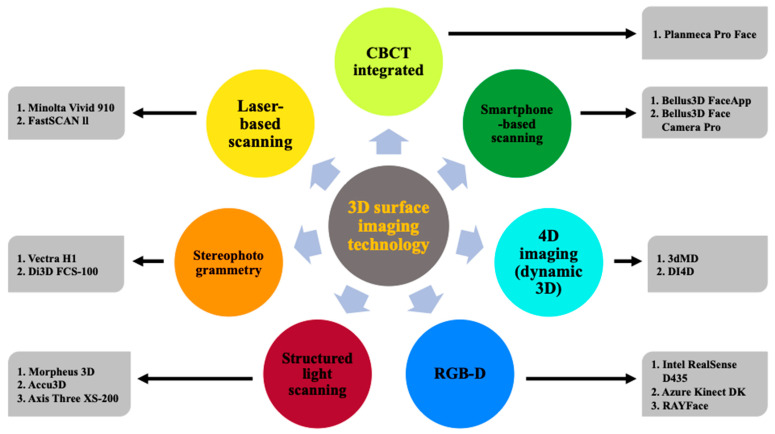
Representation of the 3D surface imaging technologies and systems: 3D, three-dimensional; CBCT, cone beam computed tomography; 4D, four-dimensional; RGB-D, red-green-blue-depth.

After removing nine duplicates, the titles and abstracts of 917 articles were screened. Of these, 836 articles were found to be irrelevant to the topic and were excluded. Following the initial screening, 149 articles (81 from the database search and 68 from additional sources) were retrieved as full texts. After a detailed review of these articles, 78 studies were eliminated. Finally, two reviewers (D.A. and P.S.) considered 71 pieces of literature that met the inclusion criteria to be suitable for qualitative analysis and included them in this review. Articles were categorized depending on the 3D visualization principle of the surface imaging system employed. Figure 3 provides a broad classification of the 3D surface imaging systems analyzed in the present review.

### 3.1. Findings on 3D Surface Imaging Technologies and Systems

The hardware, software, and functionality characteristics of the 3D face acquisition systems are summarized in Table 2. Furthermore, Table 3 highlights the limitations and drawbacks associated with each system.

#### 3.1.1. Laser-Based Scanning

The first 3D laser scanning system for clinical use was introduced in 1991 by Moss et al. to monitor the growth patterns of children with facial deformities [20]. Since then, laser scanners have been extensively used in anthropometric studies and applied clinically. They generate 3D images of the facial surface by projecting an eye-safe class 1 laser beam onto the patient’s face, which scatters the beam and determines the 3D coordinates (x, y, and z) of surface points [21,22]. The scanning process takes about 10 s and captures involuntary facial movements more distinctly than stereophotogrammetry [23]. Laser scanners for 3D imaging can be classified as single-point or slit scanners based on the beam source. For facial morphology analysis, slit scanners are a more practical option in terms of scanning time and mechanical simplicity [24]. The two most common laser scanners used for dental facial imaging are *Minolta Vivid 910* and *FastSCAN II* [25].

##### *Minolta Vivid 910* 

Minolta Vivid scanners (Konica Minolta Sensing Inc., Tokyo, Japan; https://www.konicaminolta.com/instruments/download/instruction_manual/3d/index.html) (accessed on 8 December 2023) are widely used scanners for medical or facial scanning. The currently available models include the Vivid 900 and Vivid 910 [26]. The non-contact Vivid 910 scanner captures 3D data using triangulation [27] and manifests high-speed scanning capabilities, with the ability to capture an angular field of view (FOV) of 10 cm^2^–1 m^2^ in just 2.5 s. The generated polygonal mesh contains over 300,000 vertices (connected points). The generated polygonal mesh retains all the connectivity information, thus improving the detailed capture and eliminating geometric ambiguities. Vivid 910 can automatically detect the optimal measurement distance and laser intensity through autofocus (AF) and autoexposure (AE) technologies, respectively. It can measure objects of various sizes with three interchangeable lenses and is equipped with a high-accuracy calibration unit for 3D data calculation. It can be operated without a host computer by recording data on a compact flash memory card. The acquired images are 24-bit color, without parallax errors, and oriented on the same optical axis as the 3D data, allowing for the creation of true-color 3D models.

**Validation and Dental Applications:** The *Vivid* scanner can assist in fabricating orthodontic appliances, anthropometric measurements, prosthetic and orthotic manufacturing, rapid prototyping, and forensic modeling. Kau et al. evaluated the reliability of this system for measuring facial morphology and found no significant difference between the facial scans at baseline and at 3 min and 3 days postoperatively [28]. Kovacs et al. compared manual measurements on dummies using *Vivid 910* and reported an error range of <2 mm for >93% of the data [29]. However, the accuracy was questionable because of the absence of voluntary and involuntary movements in dummies.

##### *FastSCAN II* 

*FastSCAN II* (Polhemus, Colchester, VT, USA; https://polhemus.com/scanning-digitizing/fastscan/) (accessed on 10 December 2023) is an ultra-portable, fast, and convenient laser scanner that takes less than 1 min to scan a human face. It uses a handheld wand mounted with a laser to triangulate the 3D object and a camera to record cross-sectional depth profiles, thereby measuring 3D shapes instantly by simply sweeping the wand over the object [30]. It is a dynamic system that allows for the turning and rotation of the objects during scanning. Furthermore, it is equipped with a magnetic tracking system that tracks the wand’s location and allows for the automatic registration and real-time transformation of a single scan into a 3D model. It has a sensing range of 3–6 feet and can even scan complex organic shapes that are otherwise difficult to scan. The salient features of *FastSCAN* include: (a) auto-stitching of 3D images in real time, which eliminates post-processing errors; (b) surface editing, which allows raw scan modification through the selection and deletion of raw data points; (c) on-screen direct linear measurements; and (d) accurate scanning through glass by means of mitigating refractive error. 

**Validation and Dental Applications:** *FastSCAN II* has been reported to be a reliable, accurate, and clinically valid alternative to manual measurements and a quick, easy, and useful tool for the objective assessment of craniofacial malformations [31] that can be used for manufacturing orthotics and prosthetics, rapid prototyping, and forensic modeling.

#### 3.1.2. Stereophotogrammetry

Photogrammetry is the science of photographic measurements, and involves the reconstruction of two-dimensional (2D) and 3D structures from photographic reproductions. It has been used in the fields of medicine and dentistry since the 1940s [32]; however, its first clinical use in orthodontics was reported by Thalmann-Degan in 1944 [33]. A modified and standardized version of this technique is “stereophotogrammetry”, wherein two or more stereo-paired cameras coordinate to capture an image simultaneously from different viewpoints and combine the multiple views into a 3D image [34,35,36]. Stereophotogrammetric image acquisition can be performed actively or passively, depending on whether the projected light is patterned or not. Active stereophotogrammetry involves projecting a patterned light onto an object and utilizing two or more cameras to capture the deformation in the pattern caused by the object’s surface via triangulation and calibration. This method is simpler than passive stereophotogrammetry, which requires two or more cameras in order to determine the 3D surface of an object without using a pattern projection, making the determination of correspondence between the views difficult and ambiguous in passive stereophotogrammetry. Contemporary digital stereophotogrammetry provides quantitative mesh information in the form of a dense polygon mesh accompanied by a qualitative, life-like rendering of the facial surface. High-velocity acquisition of the entire face (almost 360°) simultaneously from different viewpoints and reproduction of realistic facial surface geometry, texture, color, precision, and reproducibility render stereophotogrammetry the gold standard for facial scanning [37]. This study discusses the three commonly used stereophotogrammetry systems in facial morphology imaging.

##### *Vectra H1* 

**Figure 4:**
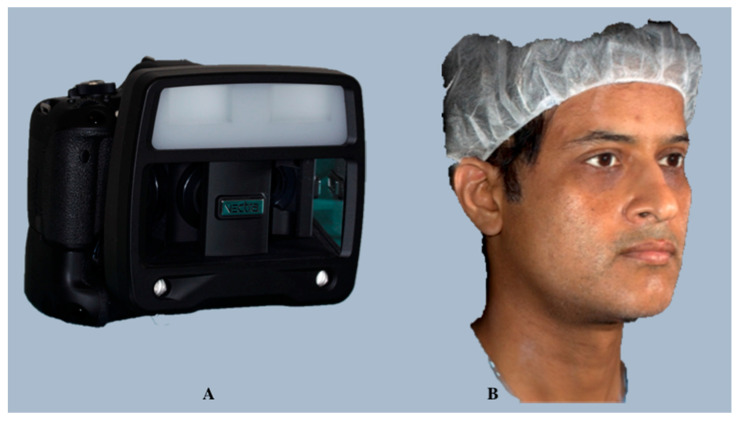
Vectra H1 facial imaging system (**A**) and an automatedly stitched 3D facial scan generated using the Vectra H1 face acquisition system (**B**).

*Canfield* (Canfield Scientific, Inc., Fairfield, NJ, USA; https://www.canfieldsci.com/) (accessed on 12 December 2023) offers several 3D surface-imaging solutions, including Vectra H1, Vectra M3, Vectra XT, and Vectra CR. Among these, passive stereophotogrammetry-based Vectra H1 has been the most favored portable imaging system as it is capable of capturing frontal faces through 100° and is affordable (Figure 4). 

*Canfield Sculptor*^TM^ software (version 6.10) enables tissue simulations with 3D surface images and has an array of striking features: it is lightweight, handheld, simple, and intuitive image capture, and can achieve automated stitching of three facial captures into one 3D image. It uses the grey mode for facial contour evaluation, and it can differentiate between red and brown skin components for skin condition assessment using *Canfield’s* proprietary *RBX^®^* technology. 

**Validation and Dental Applications:** *Vectra H1* offers 3D assessment and simulation tools, such as marker-less tracking of soft tissue changes and volumetric changes after orthognathic surgery and automated linear and angular measurements for orthodontic treatment planning. It simulates the effects of combining complementary procedures on the treatment outcome, thus facilitating easy communication of surgical and non-surgical procedures to the patients. The accuracy and reproducibility of this system have been validated in previous studies and found to be sufficiently accurate for clinical use, with a random error < 1.5 mm for linear, angular, and surface area measurements [38,39], and the average participant and technical errors have been found to be 0.40 ± 0.06 mm and 0.34 ± 0.13 mm, respectively [40].

##### *Di3D FCS-100* 

*Di3D* (Dimensional Imaging Ltd., Glasgow, Scotland; https://di4d.com/) (accessed on 14 December 2023) is another passive stereophotogrammetry-based imaging system developed by *Dimensional Imaging* in 2002. Its *Di3D FCS-100* model was specifically designed to capture 3D surface images of human faces [41]. Utilizing standard digital still cameras with the shutter speed set at 1/50th of a second and F/20 aperture, the cameras capture frontal faces instantaneously through 180º within the flash illumination (0.00125 s), thereby eliminating motion artifacts. This instantaneous capture is specifically useful for adults or children who find it difficult to stay still while capturing facial expressions. A continuous point cloud that is converted into a mesh represents the 3D geometry in *Di3D*. The company’s proprietary software, *Di3Dcapture*, integrates image capture and 3D processing automatically to generate a dense range map image, which is then merged with the original color image to produce seamless, high-definition, photographic-quality 3D surface images. The trademarked *Di3Dview* software (version 3.9) features tools for advanced 3D model alignment, mesh conformation, re-texturing, and morphing and allows for simple and effective landmark placement, point measurement, volume measurement, and symmetry assessment. 

**Validation and Dental Applications:** Khambay et al. reported a mean system error of 0.2 mm for facial casts [42], while Winder et al. reported a geometric accuracy of 0.057 mm, a reproducibility error of 0.0016 mm, and a mean error of 0.6 mm for linear measurements using the *Di3D* system [43]. Fourie et al. also confirmed the system’s accuracy and reliability for clinical and research purposes using indirect anthropometric measurements from *Di3D*-derived 3D soft tissue surface models of cadaveric heads [44]. 

#### 3.1.3. Structured Light Scanning

Structured light scanners are based on structured light patterns that capture 3D information [45]. A fully structured light pattern, such as an elliptical pattern or random texture map is projected onto the patient’s face, and the distortions in this pattern caused by the facial morphology are detected using a charged-coupled device. Following this, the distance of each point in the pattern is automatically calculated, and a 3D image is generated [46]. This technique virtually inhibits any possible motion artifact owing to its rapid capture speed, which is just a few milliseconds. 

##### *Morpheus 3D* 

**Figure 5:**
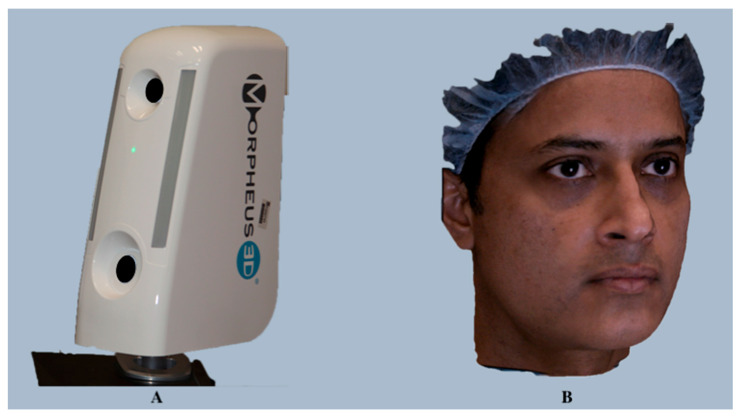
Morpheus 3D facial imaging system (**A**) and a sample of a single composite 3D facial image generated using the Morpheus 3D face acquisition system (**B**).

*Morpheus 3D* is a photogrammetry device (Morpheus Co., Ltd., Seoul, Republic of Korea; https://www.morpheus3d.co.kr) (accessed on 16 December 2023) that employs the structured light scanning principle. It uses an LED and a 3D scanner to generate 3D images and a 3D simulator that provides treatment simulations based on scanned data. This compact and user-friendly device (390 mm × 140 mm × 240 mm) captures three images (front, right, and left) from three different angles (45°) within a fraction of a second (approximately 0.8 s), and the acquired 3D image data is merged into a single composite 3D facial image within 2 min through the registration and integration processes [47] (Figure 5).

The overlapping areas resulting from the merging of the images are automatically deleted during these processes. The proprietary software, *MAS (Morpheus3D Aesthetic Solution)* (version 3.0), can perform various operations, including 3D diagnostics, patient data management, and 3D computed tomography (CT) bone surgery, thus eliminating the need for individual software for each function. 

**Validation and Dental Applications:** *Morpheus 3D* provides a complete solution for facial diagnosis via automated landmark detection; assessment of the patient’s proportion ratio and golden ratio; evaluation of facial asymmetry; linear, curved, and volumetric measurements, comparison of the pre- and post-treatment changes; and simulations for various facial treatments such as Botox, laser, thread, filler, facelift, and double eyelid surgery. Previous studies have reported good congruence with anthropometric measurements, with mean differences of 0.9 mm [48] and 0.75 mm in the accuracy of 3D images, respectively, and validated its usefulness in clinical settings. Lee et al. validated this system for facial skin thickness estimation [49], while Traisrisin et al. reported *Morpheus 3D Facemaker* software (version 3.0)to be accurate in predicting soft tissue changes following orthognathic surgery [50].

##### *Accu3D* 

**Figure 6:**
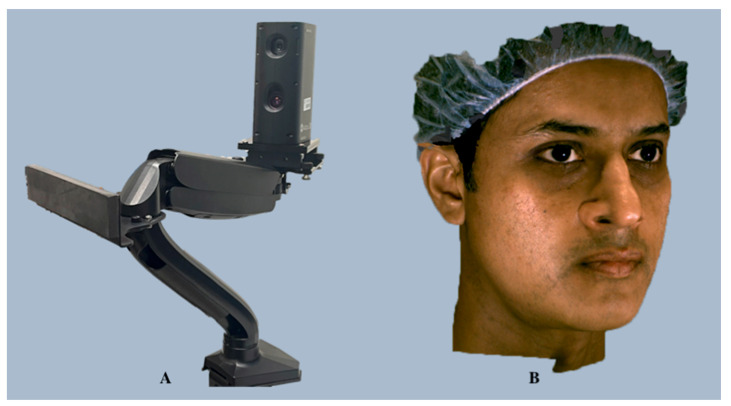
Accu3D facial imaging system (**A**) and an automatedly merged 3D facial scan generated using the Accu3D face acquisition system (**B**).

*Accu3D* (Accu3DX Co., Ltd., Tai Chung City, Taiwan; https://www.accu3dx.com) (accessed on 20 December 2023) is an ergonomically designed, portable, and user-friendly scanner with ultra-fast capture speed that delivers highly precise 3D geometry and texture quality in just 0.5 s. The system can capture the patient’s 3D image, including posteriorly located landmarks, by using an adjustable mount that can rotate beyond 180°. It offers three different scanning modes, including only face, face and neck, and all face, depending on the clinical requirement. For instance, in “all face” mode, frontal, left, right, and neck views are merged together to generate a complete 3D image (Figure 6). 

The company’s *Accu3DX Pro* software integrates web-based data management and artificial intelligence (AI)-based facial analysis technology. The intuitive web database and web viewer features provide triple encryption for patients’ 3D data that can be used on any platform worldwide. Moreover, AI-driven landmark detection and head positioning shorten pre-analysis preparation times, while a patented treatment simulation algorithm yields a treatment plan within 30 s. 

**Validation and Dental Applications:** The 3D data generated from the *Accu3D* scan can be used for orthodontic treatment planning, analyzing facial proportions, and assessing facial asymmetry. Additionally, *Accu3DX Pro* offers effective face comparison and superimposition features for pre- and post-treatment follow-ups and growth change evaluations. The scientific validity of this system is currently questionable due to a lack of peer-reviewed studies, although the company claims an accuracy of 0.2 mm.

##### *Axis Three XS-200* 

The *Axis Three XS-200* is a structured, light-based scanner developed in 2002 (Axis Three, Belfast, Ireland) which is specifically optimized to capture facial topology and simulate facial surgery outcomes [51]. The *XS-200* is a sleek, desk-mountable unit with a minimal hardware footprint and a modular plug-and-play design that integrates three high-resolution imaging heads and a patented *Color Coded Triangulation (CCT™; Siemens* and *Axis Three* patented technology*)* algorithm to facilitate anatomically accurate 3D image capturing with an optimal processing time. Three-dimensional image geometry is delivered as a continuous cloud, which is later converted into a mesh*. Axis Three*’s Tissue Behavior Simulation *(TBS™)* engine [35] facilitates precise real-time model generation within a few seconds [52], while its face module software enables the prediction and visualization of 3D postsurgical outcomes. The facial regions can be adjusted using preinstalled fine-tuning tools, with subtle yet discernible differences. Also, the software offers six different simulation views for comparison and accurate point-to-point measurement purposes. Despite gaining traction among surgeons in over 80 countries worldwide, the company went out of business in 2014. However, *CIA Medical* (https://www.ciamedical.com/search/Axis+Three+Ltd+XS+200) (accessed on 22 December 2023) stepped in as a distributor and is now providing after-sales support.

**Validation and Dental Applications:** *Axis Three XS-200* allows for the precise 3D simulation of various facial cosmetic surgery procedures, including facelifts (https://www.bodysculpt.com/face/face-lift/) (accessed on 22 December 2023), rhinoplasty (https://www.bodysculpt.com/face/) (accessed on 22 December 2023), chin implants, neck lifts, and cheek augmentation, thereby redefining the esthetic consultation experience and delivering unprecedented patient confidence and postsurgical satisfaction. While the company claims a system accuracy of approximately 0.5 mm, scientific studies have not yet been conducted to validate this claim.

#### 3.1.4. Cone-Beam Computed Tomography Integrated

##### *Planmeca ProFace* 

**Figure 7:**
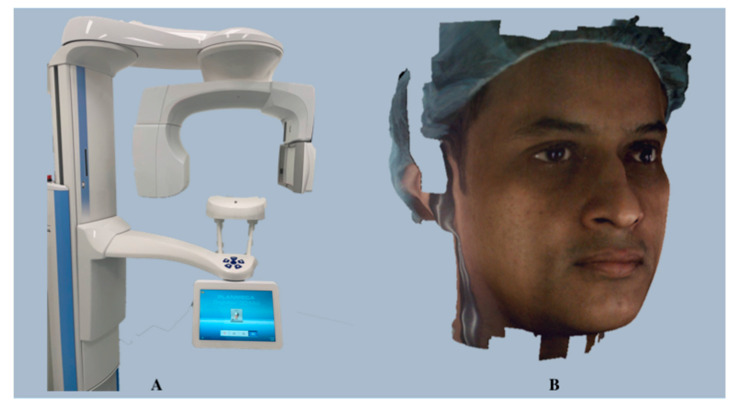
Planmeca ProFace imaging system (**A**) and a 3D face photo generated using the Planmeca ProFace imaging system (**B**).

Cone-beam computed tomography (CBCT) has been extensively commercialized and widely used in dentistry since the 1990s [53]. CBCT systems have evolved considerably over time, and several hardware and software solutions are currently available. One such update in the CBCT system by Planmeca Oy, Helsinki, Finland (https://www.planmeca.com) (accessed on 24 December 2023), is the integration of a 3D face camera. *Planmeca ProFace* is an exclusive 3D face photo system available with all *Planmeca ProMax 3D* CBCT units. It is a laser scanning-based system wherein the lasers perform facial geometry scanning and the color and texture of the face are captured by digital cameras. Additionally, it provides the freedom to generate a 3D image solely without exposing the patient to ionizing radiation. This unique system integrates CBCT volume with realistic 3D face photos in a single imaging session without any additional steps for 3D face photo acquisition [54], thus ensuring perfectly compatible images, as the patient’s position, muscle position, and facial expressions remain unchanged. The company’s copyrighted software, *Planmeca Romexis* (version 6.4.5), coalesces the acquired information into a 3D face photo (Figure 7) that can be viewed as an image or in conjunction with the CBCT volume for a detailed facial anatomy view. 

**Validation and Dental Applications:** *Planmeca ProFace* software (version 4.5.0.R) provides a safe, fast, precise, and effective tool for preoperative planning and follow-up for cosmetic, orthodontic, and maxillofacial surgeries. It allows for the visualization of soft tissues relative to the facial bones and dentine and offers various treatment planning functions, such as measurements, pre- and post-operative comparison, adjustments, and superimposition of images. The scientific validation of this system is a subject of debate. While Liberton et al. found its validity to be comparable to other surface imaging systems [55], a recent study by Amornvit et al. reported lower scanning accuracy [56].

#### 3.1.5. Smartphone-Based Scanning

##### *Bellus3D* 

Bellus3D, Inc. (Campbell, CA, USA; https://www.bellus3d.com) (accessed on 21 March 2021) is a Silicon Valley startup established in 2015 that has been a pioneer of several face scanning programs, such as the *ARC system*, *Dental Pro*, *Face Maker*, and *FaceApp* [57]. Among these, their proprietary applications, *Bellus3D FaceApp* for iOS and *Face Camera Pro* for the Android and Windows platforms, have recently been favored. Both products have been designed for self-scanning and can generate a complete 3D facial reconstruction (from left to right ear) in a single scan within a significantly short scanning time. The generated 3D scans are not only readily downloadable and can be utilized for direct printing without any post-processing, but they can also be exported to other applications and artificial reality/virtual reality digital environments.

1. *Bellus3D FaceApp*

**Figure 8:**
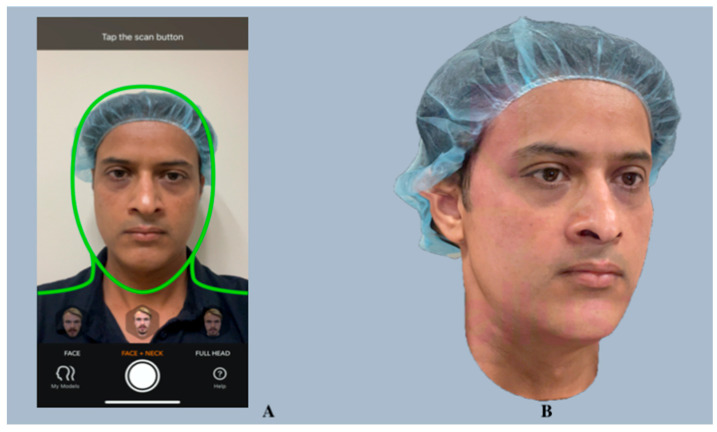
Bellus3D FaceApp interface (**A**) and a 3D facial scan generated using Bellus3D FaceApp (**B**).

*Bellus3D FaceApp* (version 3P) is a free-to-use face-scanning mobile application (app) for iPhones and iPads. *Bellus3D* was the first to utilize the iPhone or iPad’s built-in true-depth camera, which uses infrared light to project over 30,000 dots, for generating high-resolution 3D facial scans [58]. This simple, user-friendly app is capable of capturing more than 250,000 3D data points of a user’s face within 10 s. Three-dimensional face scan acquisition involves facing the smartphone’s front camera and turning the head from left to right according to the app’s voice instructions. A 3D face is then virtually constructed with lifelike quality and can be zoomed, rotated, and viewed in 3D (Figure 8). 

*FaceApp* offers options to scan the face, face and neck, or the full head, with an additional step needed for the latter two (turning the user’s head up and down). The app also employs the smartphone’s built-in gyro for controlled viewing of the 3D face, which can be saved to photo albums or shared on social media platforms. In addition, the app allows users to adjust the mesh smoothness and choose different scanning modes, such as low-definition (LD), standard-definition (SD), and high-definition (HD).

2. *Face Camera Pro*

Unlike *FaceApp*, *Face Camera Pro* is a universal serial bus (USB) accessory camera specifically developed for Android and Windows devices. It is an easy-to-use, affordable, dual-structured light scanner that should be attached to a cellphone or tablet and controlled by the company’s proprietary software program, *Face Camera App*. The camera incorporates two infrared sensors (1 megapixel; 1280 × 800 pixels), one color sensor (2 megapixels; 1600 × 1200 pixels), and two infrared laser-structured light projectors. It utilizes the *DepthShape* and *PhotoShape* technologies to capture more than 500,000 3D facial data points in one scan. Furthermore, similarly to *FaceApp*, *Face Camera Pro* requires the mere turning of the user’s head from left to right to generate amazing 3D realistic facial models within seconds that can be saved in the SD or HD scanning modes. 

**Validation and Dental Applications:** *Bellus3D* scanning platforms can be used to model and compare dental or plastic surgery outcomes in 3D. Regarding scientific validity, the mean precision and mean trueness values of *Face Camera Pro* were reported to be 0.32 mm and 0.91 mm, respectively [59], in Piedra-Cascón et al.’s study. Likewise, another study by *Cascos* et al. reported mean accuracy values of 0.61 mm and 0.28 mm with *Face Camera Pro* in maximum intercuspation and smile, respectively [60]. On the other hand, *Bellus3D FaceApp* has been reported to be less accurate than other scanners for depths greater than 2 mm. Similarly, Dzelzkaleja et al. demonstrated that the scanning results of *Bellus3D FaceApp* were not up to the standards set by other scanning apps such as Heges and Scandy Pro [61]. 

#### 3.1.6. Four-Dimensional Imaging (Dynamic 3D)

Technological advancements from 3D scanning to four-dimensional (4D) scanning systems have enabled scanning in motion and have overcome the challenges associated with 2D still and dynamic face recognition systems, such as makeup, pose variation, and illumination [62,63,64]. In this regard, *3dMD* and *DI4D* are the two commercially available 4D systems for facial scanning based on the “multi-view stereo acquisition technique” [65]. This technique employs the placement of multiple cameras at different viewpoints to capture various images of the scene, providing the corresponding points for image reconstruction. 

##### *3dMD* 

**Figure 9:**
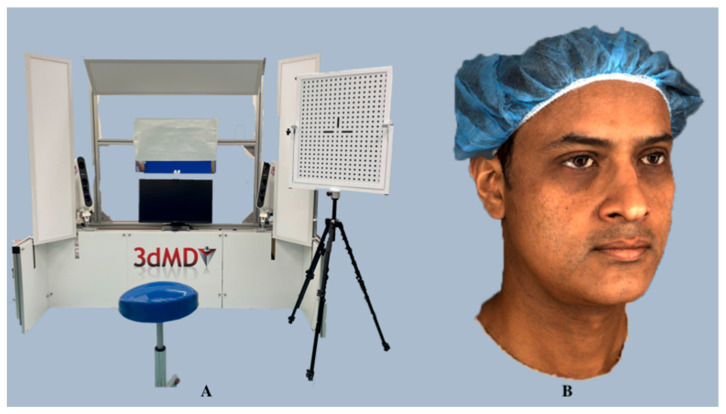
3dMDface imaging system (**A**) and a 3dMDface system-rendered 3D facial scan with accurate surface texture and color (**B**).

*3dMD* is a hybrid stereophotogrammetry system first developed in 1997 (3dMD LLC, Atlanta, GA, USA; https://3dmd.com/) (accessed on 24 December 2023), and employs both active and passive stereophotogrammetry strategies that capture the deformation of the target object’s surface in a frame-by-frame manner. Superior-quality 3D images are generated by close synchronization between machine vision cameras, high-quality sensors, and robust light-emitting diode (LED) lighting, assisted by software algorithms that are based on projected random patterns (active) as well as skin textures (passive) for triangulation. *3dMD*’s proprietary engine integrates information from all the camera viewpoints per frame, eliminating the need for manual stitching and registration*,* and automatically applies high-texture maps and renders colorful 3D images (Figure 9). 

*3dMD* technology offers 4D motion recording as a standard feature with all its products. It offers seven different preconfigured dynamic-4D capture systems, namely, *3dMDface/3dMDtrio*, *3dMDhead*, *3dMDhand*, *3dMDbody*, *3dMDfoot*, and also a customizable *3dMDflex*, all powered by *3dMD* software; however, only *3dMDface* and *3dMDtrio* have been preferred for facial surface imaging purposes. *3dMDface* uses two modular camera units (MCUs) employing six high-frame-rate machine vision cameras placed at two viewpoints and synchronized with robust LED lighting, while *3dMDtrio* comprises three MCUs that incorporate nine high-frame-rate machine vision cameras placed at three viewpoints. *3dMDface* provides 190° full face coverage and captures the face and neck from ear to ear, which can be enhanced to 220° using *3dMDtrio* and up to 360° using high-end models. *3dMD* is capable of capturing 10 min of sequential 3D surface images and enables the operator to choose the entire image sequence for analysis or render the optimal time for immediate evaluation. The geometry is represented by a continuous point cloud per frame, and thousands of individual surface points can be tracked with six degrees of freedom using dense surface tracking. Furthermore, *3dMD*’s eye-safe optics-based technology provides comfortable subject illumination even after prolonged sessions.

**Validation and Dental Applications:** A wide range of facial expressions, smiles, functions, and speech can be recorded in 4D using the *3dMD* systems. The precision, reproducibility, and accuracy of the *3dMD* have been extensively validated in the literature, making it the gold standard in 3D surface imaging [35,36,66,67], with an average technical error of 0.35 ± 0.14 mm and a mean global error of 0.2 mm reported [68] for *3dMDface* images. The system has been widely used for facial asymmetry assessment [69], evaluating sexual dimorphism [70], investigating oral and maxillofacial surgery treatment outcomes [71], obstructive sleep apnea prediction [8], anthropometric comparisons [21,67,72,73,74], assessment of facial swellings, and changes in volume [75]. Additionally, it has been utilized for developing a dynamic facial expression database [76,77], defining alar mobility [78], and biometric identification [79]. 

##### *DI4D* 

Dimensional Imaging Ltd. (Glasgow, Scotland; https://www.di4d.com) (accessed on 26 December 2023) offers a 4D capture system called *Di4D.* It is a passive stereophotogrammetry system that uses three or more video cameras to generate a complete 3D video sequence of a moving object in color. The standard configuration comprises a stereo-pair of four synchronized monochrome grayscale cameras, a stereo-pair of two synchronized color cameras (*Model avA* 1600–65 km/kc, resolution 1600 × 1200 pixels; Kodak sensor model KAI-02050, Basler, Germany), and two illumination units (Model DIV- 401-DIVA LITE; Kino Flo Corp., Burbank, CA, USA). The grayscale digital cameras capture the video sequences, while the color cameras capture the surface texture. Each frame is treated as a separate stereo-pair of images that are processed automatically to generate 3D dynamic facial images over time, which are then coalesced to produce a 4D sequence. The cameras, while working in tandem, provide facial coverage of approximately 180° and capture 3D dynamic facial images at a temporal resolution of 60 frames/s. A continuous point cloud per frame, which is converted to a consistent mesh by employing texture maps and dynamic normal maps, represents the generated geometry. Furthermore, with the introduction of next-generation, unrivaled 4D facial capture systems, such as *Di4D Pro* and *Di4D HMC*, authentic facial expressions, nuances, and subtle facial movements can be translated to highest-fidelity colored 4D facial performance data, which may have clinical applications in the near future.

**Validation and Dental Applications:** Although *Di4D* promises a wide range of possibilities for dental applications, including facial expressions, functions, smiles, and facial animations, the literature corroborating this is sparse. Algha et al. investigated the reproducibility of facial expressions that can be reliably used to quantify facial muscle movements in patients with facial palsy, and reported *Di4D* to be a viable clinical tool for the assessment of facial expressions [80]. Similarly, Shujaat et al. evaluated the feasibility of measuring the changes in the magnitude, speed, and motion similarity of facial animations in head and neck oncology patients and described *Di4D* as a reliable, practical, and feasible method for capturing the dynamic facial soft tissue movements [81]. 

#### 3.1.7. Red-Green-Blue-Depth (RGB-D)

In the clinical setting, it may be challenging to incorporate traditional facial scanners for clinical facial assessment. Therefore, consumer-grade 3D scanning alternatives, such as RGB-D (red-green-blue-depth) sensors, which combine red, green, and blue (RGB) data with depth information (D) [82], have been developed and are now being utilized in a variety of facial applications, including face detection, face authentication, face identification, and face expression recognition [83]. RGB-D sensors are compact, portable, and inexpensive and are capable of capturing 4D data, although at lower 2D and 3D resolutions. These sensors are laser-based time-of-flight (ToF) depth sensors that project infrared points onto an object’s surface, and the distance of the point is determined by the time taken by the wave to reflect and reach the scanner’s sensor to generate a 3D image. The *Intel RealSense D435 Camera* and *Azure Kinect* are among the most popular RGB-D sensors.

##### *Intel RealSense D435 Camera* 

*Intel RealSense* technology (Intel Corporation, Santa Clara, CA, USA; https://www.intel.com/content/www/us/en/homepage.html) (accessed on 28 December 2023) is a depth perception and tracking technology designed for a wide range of *Intel RealSense D400* series (*D415*, *D435i/D435*, and *D455*) depth cameras. These cameras are the leading 3D depth-sensing cameras available [84], using a depth algorithm that allows for more precise and longer-range depth perception; however, only the *D435i* and *D435* are equipped with RGB-D cameras. *RealSense D435* (https://www.intelrealsense.com/depth-camera-d435/?wapkw=Intel%20RealSense%20D435%20Camera) is a portable imaging device comprising three core elements: *Intel RealSense Module 430 D* (1280 × 720 pixels) with wide infrared projector and left and right imagers, a 2-megapixel RGB camera (1920 × 1080 pixels), and *Intel RealSense Vision Processor D4* (https://www.intel.com/content/dam/support/us/en/documents/emerging-technologies/intel-realsense-technology/Intel-RealSense-D400-Series-Datasheet.pdf) (accessed on 28 December 2023). The non-visible static infrared pattern projected by the projector improves depth accuracy in scenes with low texture, while the scene data are captured by left and right imagers and processed by the vision processor to generate a depth frame. The subsequent depth frames then create a depth video frame. The active infrared stereoscopic depth-technology-based *D435* camera offers a wide FOV for object recognition and is equipped with global shutter image sensors that provide excellent low-light sensitivity. In addition, the RGB rolling shutter sensor technology enables quality depth for an array of applications at a speed of 30 frames/s. This lightweight and powerful camera, accompanied by highly customizable software, provides low-cost sensing solutions for indoor and outdoor usage.

**Validation and Dental Applications:** Although there is little research on the reliability of the Intel RealSense D435 camera, it has various applications, including facial feature tracking and recognition, expression recognition, gesture recognition, head orientation detection, face recognition, and function recording in Bell’s palsy patients. Furthermore, it can perform face landmark tracking and skeleton tracking despite impediments such as facial hair, piercings, and eyeglasses, enabling the computation of patient movement parameters and joint angles. 

##### *Azure Kinect DK* 

The *Azure Kinect* development kit (Azure Kinect DK, Microsoft Inc., Redmond, WA, USA), released in 2020, targets users in robotics, healthcare, retail, and manufacturing. It is based on a continuous-wave ToF camera, where objects in the camera’s FOV backscatter the light projected from an amplitude-modulated light source, and the phase difference between the emitted and reflected light is measured. This phase difference is then translated into a distance value for each pixel in the imaging array. The hardware employs a 12-megapixel RGB color camera (4096 × 3072 pixels), a 1-megapixel ToF depth camera (1024 × 1024 pixels), an inertial measurement unit, and a seven-microphone circular array (https://docs.microsoft.com/en-us/azure/kinect-dk/hardware-specification) (accessed on 28 December 2023). In addition to featuring a global shutter for pixel-synchronized capturing, the ToF sensor provides better resolution, a wider FOV, pixel binning, and reduced power consumption [85]. This compact device offers a range of operating modes with varying frame rates, resolutions, and ranges [86]. The development kit features a software support kit for data procurement and body tracking and can integrate with Microsoft Azure’s cloud-based AI services. It also provides dynamic motion tracking through the *Azure Kinect Body Tracking* software development kit (SDK).

**Validation and Dental Applications:** *Azure Kinect* is a versatile device with a wide range of applications, including object recognition and reconstruction, gesture recognition, human–machine interaction, and medical examination. *Azure Kinect* has been found to be a viable 3D scanning solution for clinical and research applications, with a systematic error of less than 2 mm in previous studies [86,87,88,89,90,91,92,93].

##### *RAYFace* 

*RAYFace* (RayMedical, Ray Co. Ltd., Seongnam, Republic of Korea; http://www.raymedical.com/*)* (accessed on 30 December 2023) is a 3D, one-shot face scanning solution developed in 2020. This static system is based on the quick “Flash 3D scan” technology that enables the acquisition of 3D face data in 0.5 s. Unlike other scanning systems, *RAYFace* captures 3D facial data from multiple angles in a single shot while the object is stationary and generates 3D images in less than 1 min with optimal outputs. Easy and quick scanning, capturing vivid facial expressions, and enhanced face recognition technology are some of *RAYFace’s* standout characteristics. It is incorporated with nine simultaneously operating image sensors (2-megapixel RGB camera + depth camera with 550 mm × 310 mm FOV) that capture facial depth and shape, as well as automatic registration technology that generates an accurate and realistic 3D facial structure. In addition, assisted by enhanced Realistic Color Facial Reconstruction V2 technology, more precise and vivid 3D facial data can be reproduced with natural contrast and skin tones. The company’s proprietary software, the *RAYFace solution* (version 2.0), is an open platform that enables the quick integration of *RAYFace* with existing CBCT systems and the quick import of intraoral scans that are automatically aligned with the 3D face scan data. *RAYFace*’s compact and curvilinear design allows for smooth installation in a small space, eliminating the need for a dedicated room with bulky lighting and unwieldy cameras, and its 3D digital lighting technology is eye-safe, ensuring a natural smile during image capture without causing eye fatigue. Furthermore, in terms of user-friendliness, it can be easily delegated to assistants because of its easy software operation. 

**Validation and Dental Applications:** The *RAYFace* 3D technology enables clinicians to execute their clinical ideas in a multifaceted manner. The scanned virtual data can be used in various dental applications, including orthodontics, implant dentistry, and prosthetics, as well as for cosmetic dentistry procedures such as smile design and mock-up veneering. The integration of *RAYFace* with CT provides solutions for digital surgical guidance and prosthetic fabrication, providing precise diagnosis in plastic surgery and implantology. The predictive analytics offered by the software have been carefully linked with the actual treatment plan, allowing for easy monitoring and strict adherence to the devised treatment plan. The scanning accuracy of the *RAYFace* for creating digital face twins has been reported to be good, with an absolute surface discrepancy of 0.5277 when compared with the *MegaGen* and *Artec Eva* systems [94]. By integrating facial scans with CBCT, another study found *RAYFace* to be useful in identifying true mid-sagittal planes and anatomical landmarks [95]. 

## 4. Discussion

Over the past 30 years, 3D surface imaging systems have gained popularity in various medical disciplines, and their usage is evolving continuously. Owing to varied costs and hardware and software characteristics, different 3D face acquisition systems are used in various hospitals, and even among different departments within the same hospital. The majority of the previously published studies on 3D surface imaging systems are highly technical and focused on engineering aspects, making it challenging for clinicians to grasp the technicalities and usability of these devices [96]. This review discusses an array of cutting-edge 3D surface imaging systems that are currently in use or have the potential for future use in 3D face acquisition. The parameters outlined in Table 1 represent the hardware features, software characteristics, quality of the generated 3D images, and overall functionality of the 3D facial scanning systems. For clinical applicability, an ideal 3D face acquisition system must fulfill these criteria (Table 1).

### 4.1. Operational Considerations

Each system has distinct characteristics, advantages, and limitations that differentiate it from others and help to determine its clinical application. Three-dimensional face acquisition systems can be mobile or stationary and static or dynamic. While mobile systems like *Vectra H1*, *Intel RealSense D435*, and *Azure Kinect DK* are easily maneuverable around the target object due to their ultralight weight, stationary systems like *3dMD*, *DI4D*, and *RAYFace* provide standardized image acquisition due to their one-shot flash image capture. The choice between a mobile or stationary system depends on the specific purpose in a clinical setting. In order to capture the intricate details of the target object when scanning dental casts, models, appliances, and prostheses, a mobile system may be deemed necessary, as opposed to a stationary system for scanning faces. In both scenarios, the standardization of image acquisition is a prime requisite for the system to be clinically useful. Furthermore, a portable, ultra-lightweight system with a static sensor and brief scanning time would be advantageous for facial scanning applications because facial expressions and muscle activity may change from one side of the face to the other while capturing using a dynamic sensor, thus influencing the generated 3D face image. On the other hand, smartphone-based scanning systems such as *Bellus3D FaceApp* and *Bellus3D Face Camera Pro*, although possessing static scanners, still require the subject to turn their head, which changes the position of their neck muscles. This change in head position can affect the reliability of landmarks and surface texture in the neck region of the generated 3D image. 

### 4.2. Performance

#### 4.2.1. Accuracy and Calibration

Selecting the right 3D face acquisition system depends not only on the technology, but also on its clinical purpose and usage. The accuracy of the system is one of the deciding factors for clinical application when key decisions about treatment planning based on 3D surface imaging-derived landmark data are to be made. Technically, the correspondence between a virtual copy and the actual object or the similarity between the dimensions of the 3D copy and the real object determines the accuracy of the scanning system, which varies by system. Involuntary movements have been reported to interfere with image acquisition and to influence accuracy. Although previous studies have suggested acquiring right-, frontal-, and left-face images separately as in modern systems, such as *Morpheus 3D*, *Vectra H1*, and *Accu3D,* and merging them together to somewhat mitigate the impact of involuntary movements [97,98], this approach is not only time-consuming, as it requires multiple scans, but the generated 3D face image may lose accuracy due to changes in facial expressions, and muscle activity may change while capturing from one side of the face to the other, as mentioned earlier. 

The precision of a system may be limited to the landmarks within the volume captured or the coverage of the system. For instance, if the capturing of landmarks outside *3dMDface*’s capture range, which has ear-to-ear coverage, is deemed necessary, then a system upgrade to *3dMDtrio* or *3dMDhead*, with a wider image capture range, would be beneficial. Advanced stationary systems such as the *Vectra XT* and mobile systems like *Artec Eva* can be used alongside *3dMDface* in the clinical setting owing to their comparable reproducibility and accuracy [99]. Despite the fact that the next-generation mobile systems have outperformed the stationary systems in terms of portability and functionality, 3dMD continues to be the “gold standard” in 3D face acquisition due to its proven precision and accuracy [100,101]. “Calibration” is another important consideration for 3D face acquisition systems. For the majority of 3D face acquisition systems, calibration is required in order to obtain accurate results. Previous generation systems required on-site calibration through manual adjustments of the accuracy settings, and the total set-up time, including start-up and calibration time, has been reported to be as long as 20 min–1 h [102], which is inconvenient for both the operator and the patient. In contrast, handheld systems seem convenient to use, but they may require longer calibration times compared to static systems. However, newer systems, such as *Vectra H1* and *Bellus3D*, have been factory-calibrated and are available in ready-to-use configurations (Table 2).

#### 4.2.2. Scanning Time and Data Delivery

The scanning time plays a vital role in the accuracy of the system. The speed at which the scanner can capture a given object determines its capture/scanning speed; this speed depends on the type of technology utilized by the device. In a clinical setting, the speed of the scanner may significantly influence the diagnosis and decision-making process. The 3D image acquisition times of some systems, such as *Planmeca Pro Face* and *Bellus3D Face Camera Pro*, have been reported to be relatively high and may present greater noise than those of rapid capture systems in areas such as the eyes and ears. Furthermore, such lengthy approaches to 3D data collection may introduce motion artifacts and incorporate minor variations in the facial expression, thereby disrupting the accuracy of the final 3D image and making the scanning system clinically unreliable [103]. 

Providing quantifiable and incessant data based on facial measurements for the assessment of facial appearance and function is essential for diagnosing the severity of the abnormalities or determining the effectiveness of the intervention [104,105]. All currently available systems reviewed in this study achieve this purpose satisfactorily. Most of the systems require the target object to remain still when generating 3D data; in contrast, systems such as *3dMD*, *DI4D*, *Intel RealSense D435*, and *Azure Kinect* are capable of delivering the data even when the object is in motion. Capturing stationary objects becomes challenging when using systems with long scanning times that demand subjects remain still during the scanning period. This factor becomes particularly crucial when working with children, especially those with developmental delays or cognitive deficits, who may find it difficult to hold still for a lengthy scanning period. To mitigate these challenges, a short scanning period and specific instructions regarding facial expressions, posture, and lip position during image acquisition can help to alleviate errors. Advanced 3D scanners such as the *Di3D FCS-100* and *3dMDface* capture objects quickly in one shot, in just 1 ms and 1.5 ms, respectively, to eliminate motion artifacts and noisy raw data issues. In contrast, the *RAYFace* system relies on its “Flash 3D scan” technology for rapid image capturing and eliminating image distortion errors related to dynamic scanning or patient movements. This is particularly advantageous for patients who cannot restrain their movements for a long period of time, such as children and elderly patients.

#### 4.2.3. Image Quality

The quality of an image is a critical element that needs to be taken into account when selecting a 3D imaging system. Photorealistic images generated from 3D face acquisition systems allow for texture mapping, precise landmark identification, and treatment simulation. The higher the resolution, the more detailed the image, although at the cost of a heavy image file that requires a longer processing time. The system requirements for a high-resolution scanner are also high, as they require a powerful computer for image processing. The face is a complex 3D structure; hence, from a 3D face acquisition perspective, capturing even minute, intricate details of the face is indispensable and cannot be underestimated. The currently available passive stereophotogrammetry systems are solely dependent on natural patterns, such as skin pores, freckles, and scars. Therefore, high-resolution cameras are essential for pixel integration and high-quality 3D surface generation. Furthermore, careful lighting control using standardized flash units is crucial to overcome the sensitivity to illumination changes. Although low-cost RGB-D devices such as the *Intel RealSense D435 Camera* and *Azure Kinect* generate visible light color data in addition to depth, the face depth images they capture may also contain general depth value noise and undesirable holes (areas of invalid data). In this regard, deep-learning-driven face-specific deep learning depth image enhancers might offer a workable solution to efficiently enhance face-depth images [106].

Another key aspect that needs to be taken into account while using stereophotogrammetry systems is their inability to capture the eyes and cleft region accurately. The pattern of light used for 3D image construction interferes with the light reflected from the eye lenses, resulting in a concave appearance of the lenses instead of convex [107]. Additionally, the cleft region, which is often covered with saliva, reflects light, thereby producing artifacts in the final image. A more practical approach would be the use of a CBCT skull and a 3D stereophotograph-integrated fusion model that provides a photorealistic 3D representation of the patient’s face and may serve as a diagnostic tool for better treatment planning and postoperative evaluation by orthodontists and surgeons. A combination of two scanners—a stationary system for large areas and a hand-held scanner to fill in the details—could also be an alternative methodology for complex facial morphologies such as cleft lip or craniofacial syndromes. 

#### 4.2.4. 3D Software Solutions

The overall performance of the 3D face acquisition system is contingent upon the 3D software powering the system, which is capable of handling and processing all incoming data rapidly and accurately, thus providing real-time volumetric visualization and treatment simulation. Most of the currently available systems are equipped with indigenously built software to boost performance, although third-party solutions that are compatible with the hardware are also available. Passive stereophotogrammetry systems rely on either manual digitization of the landmarks directly on the patients’ faces or digitally on the patients’ scans. Although user training can help to achieve high accuracy and reliability with manual digitization [108], manual landmarking is associated with patient discomfort, is time-consuming, and may be cumbersome in busy clinical environments, leading to inaccuracies and human errors in the results [109]. Acquiring automated facial analysis approaches, such as neural networking, may help to alleviate the aforementioned concerns. For instance, Taylor et al. used the *Vectra M3* system for the automatic computation of the symmetry plane using *Procrustes* analysis in patients with facial asymmetry and reported minimal errors [110]. Their automated approach further substantiates the importance of a completely automated 3D facial assessment tool that leaves minimal scope for human error. The software-assisted analysis methods, surgical planning, and research outcomes of previous studies are generally based on and limited to the single system being used, and need validation using different 3D scanning systems. This would allow for the comparison of multicenter studies with diverse scanning systems and reveal the best-suited and most accurate surface imaging system for 3D face acquisition. 

To deliver the best results, clinicians must understand how to utilize the system to its full potential. Considering the busy schedules of clinicians, devices that are user-friendly, have minimal system requirements, do not require prior training, and provide rapid assessment results that can be clearly delivered to the patients are best suited for the clinical environment in terms of reduced consultation and decision-making times. Although stationary devices such as 3dMD provide quick and precise image acquisition, they are bulky and expensive. In contrast, portable or handheld devices, such as the *Vectra H1* and *Azure Kinect DK*, provide better operator control and can be effortlessly maneuvered up to several degrees around the face. This ensures more coverage, and would be more suitable for 3D face acquisition purposes. New smartphone applications in conjunction with TrueDepth sensors have exhibited encouraging results; however, their longer acquisition times necessitate greater operator precision and patient compliance. Further, the influence of the scanning environment on the performance of the 3D face acquisition system, as mentioned in this review, cannot be underestimated. Therefore, standard imaging conditions comprising optimal temperature settings and acceptable humidity levels should be maintained in the clinical environment for accurate imaging outcomes. Finally, several 3D surface imaging systems are currently available, with varied prices corresponding to their inherent features, data capture speed, and image quality. Their clinical usage should be based on their purpose and workflow, irrespective of their price. 

The human face is a complex 3D configuration with convexities, concavities, and tricky angles; hence, capturing its intricate details requires a comprehensive understanding of the factors described in this review. Although this review is limited to the handful of systems currently available for 3D face acquisition, they are capable of generating high-quality 3D facial images, given that the aforementioned factors are considered. 

## 5. Future Directions

Both future clinical practice and research stand to benefit greatly from the rapid development of AI technology and facial scanning. It would eventually be possible, for example, to automatically set bilaterally balanced denture teeth through the integration of an ML-guided tooth arrangement robot and a virtual articulator with a 3D facial scan. In addition, a virtual patient created from a 3D facial scan would offer enough facial landmarks to aid in planning the final prosthesis in situations where extensive full-mouth rehabilitation is planned, but there are not many landmarks for the occlusal plane. Furthermore, integration of 3D surface imaging systems with other imaging tools and 3D printing technologies could enable individualized surgical planning and simulation, treatment sequencing, patient-specific implant and prosthesis fabrication, and patient education in the future. 

The potential of 3D face acquisition technology to revolutionize healthcare is vast. Widespread adoption of 3D face acquisition for telemedicine, dermatological purposes, and facial reconstruction and forensic identification purposes is likely to enhance the quality of life. Remote monitoring of patients’ health and well-being through 3D face scanning will enable healthcare providers to provide virtual care and support to those unable to visit a healthcare facility. Automated algorithms will be able to precisely track changes in 3D facial images, allowing dermatologists to monitor disease progression and detect conditions like melanoma and skin cancer over time. Furthermore, 3D face acquisition could represent a valuable aid in facial reconstruction following trauma or deformity, as well as in forensic contexts to facilitate evidence collection and subject recognition through facial reconstruction. 

Additionally, the widespread implementation of 3D face acquisition technology for product customization, such as breathing masks and medical devices like sleep apnea masks or eyeglasses, is foreseeable. However, to fully harness the potential of 3D face scanning, the development of a handheld, versatile, scientifically validated, and cost-effective 3D surface imaging system for clinical and research purposes is highly desirable.

## 6. Conclusions

The challenge of precise facial evaluation has led to the development of contemporary 3D surface imaging systems, each with their own inherent features and limitations. This review offers updated information on these technologies and systems to assist clinicians in selecting an optimal 3D face acquisition system. Advanced 3D face scanners, powered by cutting-edge technology and sophisticated software tools, generate 3D facial images of photorealistic quality with remarkable accuracy and reliability and can be integrated with other imaging tools and modernized 3D printing technologies. While some of these systems have already been adapted in clinical settings, the results of other advanced systems seem promising. However, scientific validation of the currently available systems across multiple centers is desirable before a system can be deemed the gold standard for 3D face acquisition. Furthermore, driven by technological advances, novel devices will become cost-effective and portable, and will enable accurate quantitative assessments, rapid treatment simulations, and definitively enhanced outcomes.

## Figures and Tables

**Table 1:** Definitions of the characteristics studied in 3D face acquisition systems.

Characteristics		Definition
***Hardware***	Portability	Hand-held and compact or bulky and cumbersome to relocate
	System mobility	System is fixed or mobile while scanning
	Sensor position	Sensor is static or dynamic while scanning
	Cost-effectiveness	Inexpensive equipment and price-worthy operation to use in a clinical setting
***Software***	CT/CBCT integration	Permits integration with other imaging tools such as CT/CBCT
	Surgery simulation	Allows simulation of surgical procedures through indigenous software or third-party-assisted software
	Real-time 3D volumetric visualization	Capability to generate real-time photorealistic 3D virtual copy of the face
	Tissue behavior simulation	Predicts the post-treatment outcomes based on indigenous software or third-party-assisted software
	Progress monitoring and outcome evaluation	Enables treatment monitoring at different time points and outcome evaluation
***Functionality***	Purpose	Provision of facial measurement-based quantifiable and incessant data
	Data delivery	Delivers data while the object is still or in motion
	Scanning time	Time required by the system to scan an object
	Processing time	Time required by the software from editing and merging the acquired meshes to generating a 3D model
	Coverage	Captures only the face (excluding ears), face and neck, or full face (ear-to-ear) and neck
	Scan requisite	Requires a single scan, multiple continuous scans, or multiple scans stitched together to generate a 3D image
	Accuracy	Data generated by the system are sufficiently close to the real data
	Precision	Data generated by the system display high reliability
	Archivable data	Data generated by the system can be stored in industry standard and easily accessible formats
	User-friendly	Does not require specialized training and equipment
	System requirements	Does not have extensive hardware or software requirements

3D, three-dimensional; CT/CBCT, computed tomography/cone beam computed tomography.

**Table 2:** Hardware, software, and functionality characteristics of 3D face acquisition systems analyzed in the review.

		3D Face Acquisition System														
Characteristics		Laser-Based Scanning		Stereophotogrammetry		Structured Light Scanning			CBCT Integrated	Smartphone-Based Scanning		4D Imaging		RGB-D		
		*Minolta Vivid 910*	*FastSCAN II*	*Vectra H1*	*Di3D FCS-100*	*Morpheus 3D*	*Accu3D*	*Axis Three XS-200*	*Planmeca Pro Face*	*Bellus3D FaceApp*	*Bellus3D Face Camera Pro*	*3dMD*	*DI4D*	*Intel RealSense D435*	*Azure Kinect* *DK*	*RAYFace*
**Hardware**	*Portability*	Y	Y	Y	Y	Y	Y	Y	N	Y	Y	N	N	Y	Y	Y
	*System mobility*	Stationary	Mobile	Mobile	Stationary	Stationary	Mobile	Stationary	Stationary	Stationary	Stationary	Stationary	Stationary	Mobile	Mobile	Stationary
	*Sensor position*	Static	Dynamic	Dynamic	Static	Static *	Dynamic	Static	Dynamic	Static *	Static *	Static	Static	Dynamic	Dynamic	Static
	*Cost-effective*	Y	Y	Y	N	-	Y	Y	N	Y	Y	N	N	Y	Y	Y
**Software**	*CT/CBCT integration*	-	-	Third-party software	Third-party software	-	-	N	Romexis	N	N	3dMDvultus/third-party software	-	-	-	RAYFace solution
	*Surgery simulation*	-	-	Y	Third-party software	Y	Y	Y	Y	N	N	Y	-	N	-	-
	*Real-time 3D volumetric* *visualization*	Y	Y	Y	Y	Y	Y	Y	-	N	N	Y	Y	-	-	Y
	*Tissue behavior* *simulation*	-	-	Y	Third-party software	Y	Y	Y	-	N	N	Y	-	-	-	-
	*Progress and outcome monitoring*	-	-	Y	Y	Y	Y	Y	-	N	N	Y	-	-	-	Y
**Functionality**	*Purpose*	Y	Y	Y	Y	Y	Y	Y	Y	Y	Y	Y	Y	Y	Y	Y
	*Data delivery*	Still	Still	Still	Still	Still	Still	Still	Still	Still	Still	Motile	Motile	Motile	Motile	Still
	*Capture speed/Scanning time*	0.3–2.5 s	<1 min	2 ms	1 ms	0.8 s	0.5 s	<2 s	30 s	10 s	25 s	≈1.5 ms/1–120 fps	≈2 ms/f	90 fps	20.3 ms	0.5 s
	*Processing time*	-	-	≈20 s	60 s	<2 min	<1 min	-	-	-	15–30 s	<8 s	30 s	-	-	<1 min
	*Scan range*	1300 × 1100 mm	50 cm	≈100°	≈180°	225 × 300 mm	-	≈180°	-	-	66–69°	190°–360°	≈180°	87° × 58°	120° × 120°	550 × 310 mm
	*Coverage*	Face	Full face	Full face	Full face	Face	Full face	Face + Neck	Face	Full face	Full face	Full face	Full face	Face	Face	Full face
	*Optimal 3D measurement range*	0.6–1.2 m	2–4 inch	350–450 mm	-	650 mm	45–50 cm	1 m	-	-	30–45 cm	1 m	-	0.3–3 m	0.25–2.21 m	-
	*Color image*	Y	N	Y	Y	Y	Y	Y	Y	Y	Y	Y	Y	Y	Y	Y
	*Scan requisite*	Multiple	Multiple	Multiple	Single	Multiple	Multiple	Single	Single	Single	Single	Single	Single	Multiple	Multiple	Single
	*Output format*	stl, dxf, obj, ascii points, vrml	Several format options	-	Several format options	-	Several format options	-	stl	obj, stl	.obj, .mtl, .jpeg, .stl, .yml	Several format options	Several format options	-	-	stl, .obj
	*Scan processing software-enabled*	Y	Y	Y	Di3Dview	MAS	Accu3DX Pro	Y	Romexis	Y	Y	3dMDvultus	DI4Dlive	Intel RealSense SDK 2.0	Y	RAYFace solution
	*Accuracy*	Y	Y	Y	Y	Y	-	-	N	Y	Y	Y	Y	-	Y	-
	*Precision*	Y	Y	Y	Y	Y	-	-	N	Y	Y	Y	-	-	Y	-
	*Archivable data*	Y	Y	Y	Y	-	Y	-	Y	Y	Y	Y	Y	-		-
	*User-friendliness*	Y	Y	Y	Y	Y	Y	N	-	Y	Y	Y	Y	Y	Y	Y
	*System requirements*	Minimal	Minimal	Minimal	Minimal	Minimal	Minimal	Minimal	Minimal	Minimal	Minimal	Extensive	Minimal	Minimal	Minimal	Minimal
	*Calibration time*	NR	-	NR	5 min	-	-	<5 min	-	NR	NR	20–100 s	5 min	-	-	-

3D, three-dimensional; CBCT, cone-beam computed tomography; CT, computed tomography; 4D, four-dimensional; RGB-D, red-green-blue-depth; * requires turning the subject’s face; Y, yes; N, no; NR, not required; fps, frames per second; f, frame; third-party software such as Dolphin, Maxilim, and Materialize OMS; MAS, Morpheus Aesthetic Solution.

**Table 3:** Limitations and drawbacks of 3D face acquisition systems.

3D Face Acquisition System	Disadvantages and Limitations
**Minolta Vivid 910**	Sensitive to lighting conditions, but operates well indoors. Patients may notice a quick red flash when the laser stripe crosses the pupil. Clinical usage is limited owing to their inability to assess dynamic facial function. Slow acquisition times may introduce motion artifacts.
**FastSCAN II**	Large metal objects and electromagnetic fields may interfere with the scanner’s tracking and performance. The number of studies validating the system is limited.
**Vectra H1,** **Di3D FCS-100**	Surface texture details may be influenced by the glare on the patient’s face caused by strong directional ambient light. *Di3D* system is no longer available for commercial use, as the company has replaced it with a more advanced *DI4D* system.
**Morpheus 3D**	Occasional localized distortion of the image may occur in the integration line region. Does not support the export of the generated 3D facial images.
**Accu3D**	Additional lighting conditions are required as the reflected image on the screen appears dark, which makes it difficult to follow the image capture protocol. Patient must be seated very close to the chest rest attachment of the mount during image capture. The system has not been scientifically validated yet.
**Axis Three XS-200**	Slow capture speed of 2 s may result in missing or noisy raw data. Re-calibration is required each time the hardware is moved. The system has not been scientifically validated yet.
**Planmeca Pro Face**	Lengthy image acquisition times. The *ProFace* scanner is sensitive to subtle movements or blinking during the scanning, which may affect the image resolution around the eyes, specifically in the exocanthion region. Capturing posterior facial landmarks, including the tragus and otobasion, is challenging owing to *ProFace* scanner’s limited field of view. The *ProFace* system’s standard camera positioning may limit the coverage of landmarks underneath the chin, such as the gnathion.
**Bellus3D FaceApp** **Bellus3D Face Camera Pro**	True-depth camera’s sensitivity to infrared interference can affect the quality of 3D images in bright sunlight. *Bellus3D FaceApp* lacks conformity with the scanned face. Scanning of hair is difficult with *Face Camera Pro*. Only one 3D scan can be downloaded at a time, and the download is expensive. The company discontinued 3D face scanning operations recently, and the purchase or downloading of their items may not be feasible today.
**3dMD**	Recalibration may be required in some cases prior to image capture. May not provide a good representation of the prominent areas of the face, such as the nose, ear, and lip margins and salivated cleft regions in non-treated cleft patients. Daylight can cause lighting artifacts and affect the accuracy of generated 3D faces. The overly large letters of the pre-labeled landmarks in the software make precise on-screen marking of landmarks around the mouth and nose difficult.
**DI4D**	System is not yet validated for surgery simulation or TBS studies.
**Intel RealSense D435**	*Intel RealSense* cameras are sensitive to light intensity variances. Depth quality and resolution deteriorate as the distance of the object from the camera increases.
**Azure Kinect DK**	Poor resolution may culminate in inaccurate results. Depth measurements are sensitive to off-center object placement, optical distortions of the lens, and calibration accuracy.
**RAYFace**	Image distortion in curved facial regions such as subzygomatic and subnasal areas.

## Data Availability

The datasets used and/or analyzed during the current study are available from the corresponding author upon reasonable request due to for research purposes.
